# Cardiac magnetic resonance findings in mitochondrial disease: a guide for clinicians

**DOI:** 10.1093/ehjimp/qyaf134

**Published:** 2025-11-28

**Authors:** Rhys Gray, Vasiliki Kantartzı, Luis R Lopes, Konstantinos Savvatis, Mark Westwood

**Affiliations:** Department of Inherited Cardiovascular Diseases, Barts Heart Centre, W Smithfield, London EC1A 7BE, UK; Department of Inherited Cardiovascular Diseases, Barts Heart Centre, W Smithfield, London EC1A 7BE, UK; Department of Inherited Cardiovascular Diseases, Barts Heart Centre, W Smithfield, London EC1A 7BE, UK; Institute of Cardiovascular Science, University College London, Gower St, London WC1E 6BT, UK; Department of Inherited Cardiovascular Diseases, Barts Heart Centre, W Smithfield, London EC1A 7BE, UK; Institute of Cardiovascular Science, University College London, Gower St, London WC1E 6BT, UK; Department of Inherited Cardiovascular Diseases, Barts Heart Centre, W Smithfield, London EC1A 7BE, UK

**Keywords:** mitochondrial disease, mitochondria myopathy, mitochondrial cardiomyopathy, cardiac MRI

## Abstract

Mitochondrial myopathies are heritable conditions caused by genetic variations in mitochondrial DNA or nuclear DNA. These result in dysfunctional cellular oxidative phosphorylation and ATP production, affecting organs with high-energy requirements such as the heart, brain and skeletal muscle. Cardiac involvement is common affecting one third of patients and includes left ventricular hypertrophy, conduction disease, Wolff-Parkinson-White syndrome, and dilated cardiomyopathy. Due to the variability in the clinical presentation, a multiparametric approach incorporating clinical, biochemical, histological/histochemical and genetic criteria is required to make the diagnosis. Cardiologists should be aware of the clinical red flags and imaging findings and how to differentiate mitochondrial cardiomyopathy from other causes of left ventricular hypertrophy. Cardiovascular magnetic resonance imaging is a highly sensitive tool for depicting myocardial abnormalities to aid in both the diagnosis of patients presenting with left ventricular hypertrophy, and in the assessment of cardiac involvement in patients with a known diagnosis of mitochondrial myopathy, as this is an independent predictor of morbidity and early mortality. The most common CMRI findings include increased maximal LV wall thickness and mass and non-ischaemic subepicardial and midwall LGE, most commonly affecting the basal inferolateral or lateral wall. Future studies should consider integrating late gadolinium enhancement imaging into risk prediction models to enhance stratification of major adverse cardiac events such as heart failure and arrhythmia. As our understanding of mitochondrial disease evolves, integrating advanced imaging with molecular diagnostics will be essential for early detection of disease, improved risk prediction and outcomes.

## Introduction

Mitochondrial myopathies are heritable conditions caused by genetic variations either in the mitochondrial DNA or nuclear DNA.^1^ These result in dysfunctional cellular oxidative phosphorylation and ATP production, preferentially affecting organs with high-energy requirements such as the heart, brain and skeletal muscle.^2^ Mitochondrial DNA mutations have a maternal inheritance pattern and are the commonest cause of mitochondrial myopathy in adults.^1,3^ Genetic variations in mitochondrial or nuclear DNA are one of the most common inborn errors of metabolism, with a conservative estimated prevalence of approximately 1:5000.^4^ The clinical spectrum in mitochondrial myopathy is wide, with both isolated organ involvement and multisystem disease recognized.^3^ Mitochondrial cardiomyopathy is a common complication and can be described as abnormal myocardial structure and/or function secondary to genetic defects resulting in impairment of the mitochondrial respiratory chain, in the absence of concomitant coronary artery disease, hypertension, valvular disease, or congenital heart disease.^5^ Cardiovascular magnetic resonance imaging (CMRI) is a highly sensitive tool for depicting myocardial abnormalities to aid in both the diagnosis of mitochondrial cardiomyopathy in patients presenting with left ventricular hypertrophy (LVH), and in the assessment of cardiac involvement in patients with a known diagnosis of mitochondrial myopathy, as this is an independent predictor of morbidity and early mortality.^6^ In this review we summarize the main CMRI findings in patients with mitochondrial myopathy and discuss its utility in the clinical management of this condition.

## Clinical features

**Figure 1:**
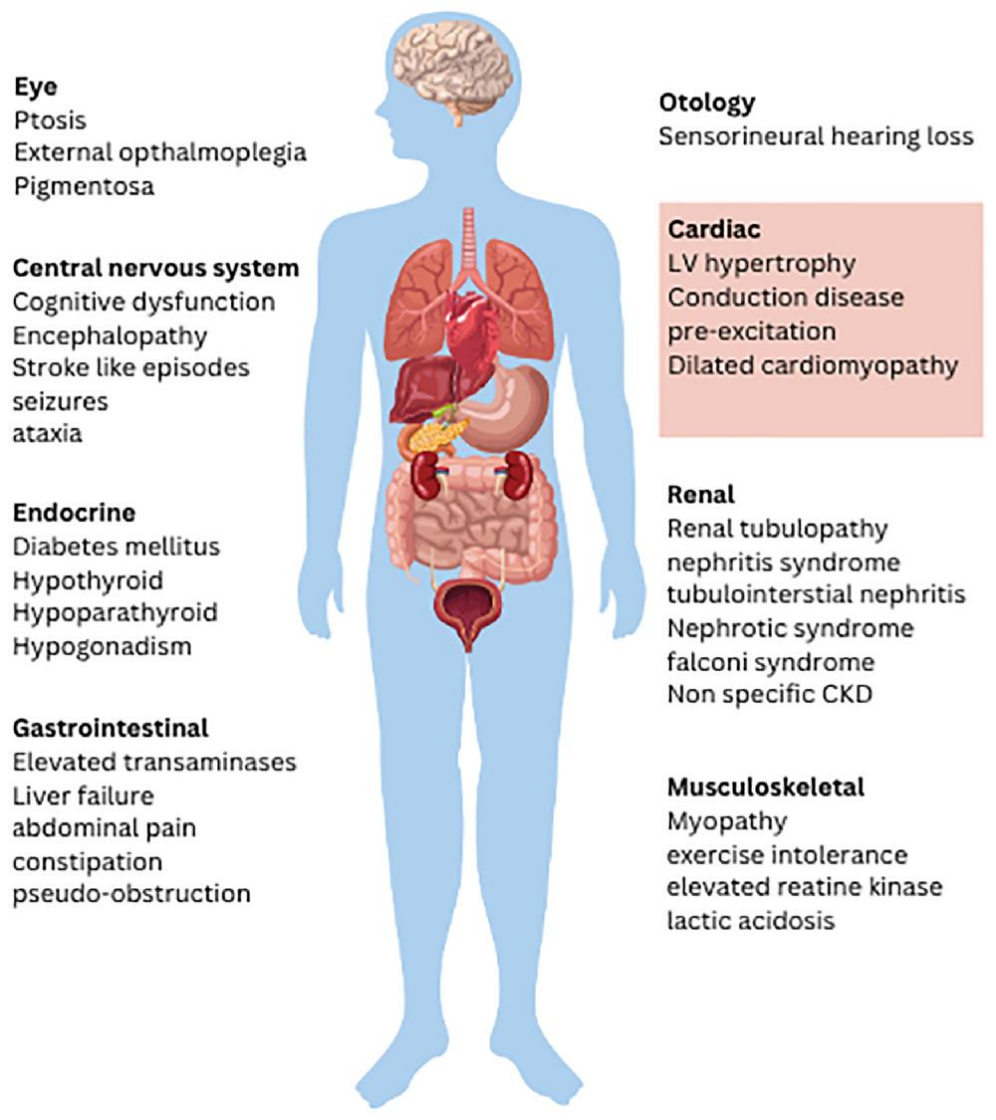
Clinical features of mitochondrial myopathy. The clinical spectrum in mitochondrial myopathy is wide with both isolated organ involvement and multisystem disease recognized.

Based on the clinical phenotype, subforms of mitochondrial myopathy have been described such as chronic progressive external ophthalmoplegia (CPEO), Kearns-Sayre syndrome (KSS), mitochondrial encephalomyopathy with lactic acidosis and stroke-like episodes (MELAS), and myoclonic epilepsy with ragged-red fibres (MERRF) among others.^7^ However, there is often considerable clinical variability, and many affected individuals do not fit into one particular syndrome.^8^ Common clinical features can include learning difficulties, sensorineural deafness, retinopathy, ptosis, dysphagia, myopathy and exercise intolerance, ataxia, diabetes, renal failure, seizures and encephalopathy (*Figure 1*). Cardiac involvement is common affecting 1 in 3 patients with left ventricular hypertrophy, conduction disease, Wolff-Parkinson-White syndrome, and dilated cardiomyopathy among the most frequently reported manifestations (*Figure 1*).^1^  ^,9–11^ Due to the extreme variability in the clinical presentation of mitochondrial myopathy, a multiparametric approach incorporating clinical, biochemical, histological/histochemical and genetic criteria is required to make the diagnosis.^12^

### Cardiac involvement in mitochondrial myopathy

Normal cardiac contractile and relaxation function are critically dependent on a continuous energy supply, and therefore the myocardium is highly dependent on oxidative metabolism.^8^ Based on the prevalence of mitochondrial disease and frequency of cardiac manifestations, about 1/10 000–15 000 of the general population are affected with mitochondrial cardiomyopathy.^3^ Early small-sized studies mainly based on electrocardiogram (ECG) recordings and/or echocardiography have revealed different forms of cardiomyopathy associated with mitochondrial disorders including both hypertrophic and dilated cardiomyopathy (*Table 1*), suggesting an incidence of cardiac involvement varying between 20–40%.^13,14^ Similarly, A recent large multi-centre study by Savvatis *et al*. of 600 adult patients with pathogenic or likely pathogenic variants in mitochondrial or nuclear genes found that cardiomyopathy based on ECG and echocardiography was present in approximately 30% of patients.^1^ Left ventricular hypertrophy (LVH) was the predominant phenotype in 23% of the cohort with 4% having a dilated LV cavity and 7% having an EF <50%. They also found 9% of the cohort had evidence of conduction disease, demonstrated by either RBBB, LBBB or first degree heart block despite the young median age of 43.^1^ A systematic review and meta-analysis of 21 studies including 825 mitochondrial myopathy patients by Quadir *et al*. found 39% of patients had conduction abnormalities on ECG and 29% had cardiac structural abnormalities with LVH being the most common finding. They found the incidence of cardiomyopathy was more prevalent in patients with the m.3243A > G gene mutation and in patients with MELAS or MERRF clinical syndromes.^15^  *Table 1* outlines the main mitochondrial disorders with known cardiac manifestations.

**Table 1:** Summary of mitochondrial disorders with cardiac phenotypes

Disease	Incidence Age at Onset	Primary Phenotype	Cardiac Manifestations	Genetic Mutations
MELAS	0.18/100 000	Stroke-like symptoms Encephalopathy Lactic Acidemia Myopathy	HCM, DCM Conduction abnormalities (WPW)	A3243G (80% of cases)
	<20 years			
MERRF	0.9 or <1/100 000	Myoclonus Lactic acidosis Cerebellar ataxia Muscle weakness Ragged red fibres	DCM HCM Conduction abnormalities (WPW, SVT, RBBB)	A8344G (83–90% of cases and 53% of cases with cardiac involvement)
	10–20 years			
MIDD	6/100 000(∼1% of patients with diabetes)	Diabetes (type I or II) Bilateral neurosensory hearing loss	LVH (55%) HCM (15–30%) Conduction abnormalities (WPW, SSS, Afib)	A3243G
	<35 years			
MNGIE	* Only 100 cases ever reported	Chronic intestinal dysmotility Leukoencephalopathy Failure to thrive Ptosis Ophthalmoparesis Peripheral neuropathy	LVH Conduction abnormalities (prolonged QT, SVT, sudden cardiac death)	Loss of function variants in thymidine phosphorylase (TP) gene, chromosome 22q13.32-qter
	Mean age of onset 18 years			
LHON	1/31 000–50 000	Acute/subacute painless vision loss Dystonia Peripheral neuropathy	LV hypertrebeculation Prolonged QT WPW	90% caused by G11778A (ND4 gene), G3460A (ND1 gene), and the T14484C (NG6 gene) which all cause dysfunction in complex I
	2–87 years			
Kearns-Sayre Syndrome	1–3/100 000	Progressive external ophthalmoplegia (ptosis, weakness of eye muscles) Retinopathy Ataxia Elevated CSF proteins	Conduction abnormalities (AV block requiring PPM) Cardiac arrest	Large deletions ranging from 1000 to 10 000 base pairs
CPEO	1–3/100 000	Loss of motor function of the eye and eyelid Spectrum of disorders with PS and KSS	HCM Arrhythmias	Large mtDNA deletions similar to PS or KSS
				AD: Variants in nuclear-encoded genes ANT1, C10orf2 and POLG
Friedreich's Ataxia	1–47:1 000 000	Progressive ataxia/areflexia Progressive and life-threatening cardiomyopathy (2/3 patients die from cardiovascular causes)	HCM DCM	Expansion of DNA triplet intron repeat GAA in the frataxin (FXN) gene
	Presents in childhood			
	Mean life expectancy 40 years			
Leigh Syndrome	1/32 000–40 000	Encephalopathy with cognitive and behavioural dysfunction Seizures Hypotonia/Ataxia Oculomotor dysfunction Respiratory dysfunction	HCM Pericardial effusions Conduction abnormalities	Variants in SURF1 gene
	<1–2 years			G13513A (WPW and HCM)
NARP	1/12 000–40 000	Neuropathy Ataxia Retinitis Pigmentosa Deafness Myoclonic epilepsy	DCM HCM Conduction abnormalities (WPW)	Point mutations at 8993
	3–12 months			
				MT-ATP6 gene (most commonly T8993G, then T8993C)
GRACILE	1/47 000 (in Finland, may be lower worldwide)	Growth restriction Aminoaciduria Cholestasis Iron overload Early death	Prolonged QT Reduced levels of complex III in myocardial tissues post mortem	homozygous point mutation A232G within the BCS1L gene
	Onset *in utero*			
Barth Syndrome	1:300 000–400 000	DCM Skeletal myopathy (proximal) Neutropenia Growth retardation	DCM Cardiac abnormalities *in utero* Endocardial fibroelastosis LV non-compaction Conduction abnormalities (prolonged WT, SVT, WPW, VT)	Point mutations or deletions affecting Nuclear DNA gene encoding for the cardiolipin transacylase tafazzin (TAZ).
	<1 year			
Pearson Syndrome	1/1 000 000	Transfusion-dependent anaemia (presenting finding) Severe Infections Liver, Kidney, Pancreas and CNS abnormalities	Increased wall thickness Depolarization abnormalities Prolonged QT	Large deletions ranging from 4.9–14 kb
	Presents in infancy			
Isolated cardiac involvement	0.6% of patients with HCM phenotype.	cardiac-only phenotype (i.e. HCM in the absence of systemic/neurological features).	HCM, extensive fibrosis and progressive remodelling to left ventricular dilation and systolic dysfunction.	m.4300A > G mtDNA variant
	Young adulthood			

### Clinical case

**Figure 2:**
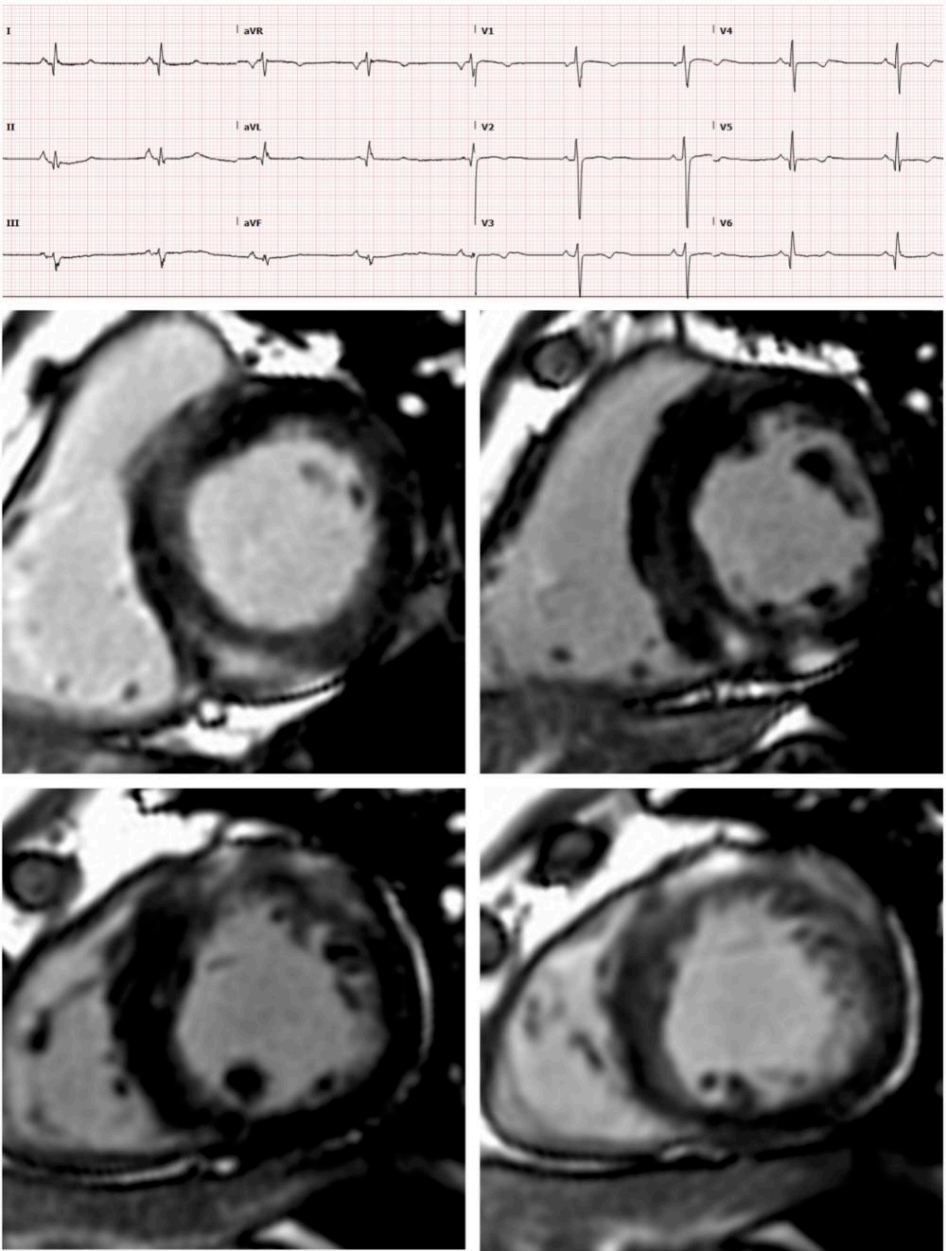
12 lead ECG and cardiac MRI late gadolinium images of a 30year old man with mitochondrial myopathy (m.3243A > G, MELAS clinical syndrome) demonstrating midwall fibrosis in the basal to mid inferior (top panel) and mid to apical anterior (bottom panel) segments.

A 30-year-old man presented to the emergency department in Singapore with severe myalgia and muscle weakness following a mountain climbing expedition. He was diagnosed with acute rhabdomyolysis and acute kidney injury, requiring hospitalization abroad for intravenous fluid resuscitation and dialysis. Renal function recovered fully over the subsequent days. The patient reported no prior episodes of muscle pain or weakness during routine activities or regular exercise and was otherwise in good health. His medical history was notable for bilateral hearing impairment. Initial electrocardiogram revealed sinus rhythm, fragmented QRS complexes in all limb leads with poor R-wave progression across the precordial leads, and anterior T wave inversion (*Figure 2*). Transthoracic echocardiography demonstrated normal left ventricular size and systolic function, with evidence of asymmetrical LV hypertrophy. A provisional diagnosis of hypertrophic cardiomyopathy was made during his admission. Upon returning to the United Kingdom, the patient was referred to the inherited cardiac conditions service at Barts Heart Centre for further evaluation. Cardiac magnetic resonance imaging revealed moderate asymmetrical LV hypertrophy (14mm) without outflow tract obstruction. There was extensive, mid-wall late gadolinium enhancement, predominantly in the basal to apical inferior and mid to apical anterior segments, sparing the septum and not in the regions of maximal hypertrophy. Although these findings could be explained by sarcomeric hypertrophic cardiomyopathy, the pattern combined with clinical red flags also raised suspicion for an underlying metabolic aetiology. (*Figure 2*). A 24-hour Holter monitor showed no significant tachy- or bradyarrhythmia. Cardiopulmonary exercise testing was abnormal with the patient only completing 6 min of exercise time, with a peak VO₂ of 18.9 mL/kg/min (47% of predicted). Repeat laboratory investigations revealed normal renal function, but elevated venous lactate at 5.8 mmol/L and an increased urine protein-to-creatinine ratio of 41 mg/mmol. HbA1c levels were markedly elevated, confirming a diagnosis of diabetes mellitus.

Given the constellation of cardiac, neuromuscular, metabolic, and endocrine findings, a mitochondrial disorder was suspected. Mitochondrial DNA analysis identified a known pathogenic variant (m.3243A > G), confirming the diagnosis of mitochondrial disease. Subsequent evaluations also revealed a moderate length-dependent axonal polyneuropathy. Over the ensuing years, the patient experienced stroke-like episodes, consistent with the progression of his mitochondrial condition and the MELAS clinical syndrome. This case illustrates the heterogeneous and often subtle presentation of mitochondrial disorders, which can lead to delayed or missed diagnosis. In this patient, cardiac imaging abnormalities, in combination with rhabdomyolysis, reduced exercise capacity, hearing impairment, elevated lactate levels, and new-onset diabetes, led to the recognition of a mitochondrial disorder that was confirmed on genetic testing.

### CMRI findings in mitochondrial cardiomyopathy

There is limited data on the CMR findings in mitochondrial myopathy patients with small retrospective cohort studies and prospective case control studies of patients mainly with either the MELAS, CPEO/KSS or MERRF clinical syndromes.

One of the earliest CMR studies by Hollingsworth *et al*. compared 10 patients with the m.3243A > G mitochondrial DNA mutation associated with the MELAS clinical syndrome to 10 matched controls.^16^ They found significant concentric left ventricular hypertrophy in the absence of systemic hypertension; and reduced global longitudinal strain in patients with the m.3243A > G gene mutation, however late gadolinium imaging was not performed. Interestingly they found that the degree of cardiac dysfunction correlated with the percentage level of mutant mtDNA in skeletal muscle on muscle biopsy.^16^ A similar study by Lindroos *et al*. compared 14 patients with the m.3243A > G gene mutation associated with the MELAS clinical syndrome with 14 matched controls and also found reduced end diastolic volume, reduced ejection fraction indexed to BSA as well as increased LV mass compared to controls.^17^ Bates at el also studied 22 adult patients with mitochondrial myopathy due to the m.3243A > G mutation, and 22 age- and gender-matched control subjects.^3^ They found significantly reduced end systolic and end diastolic volumes, significantly increased LV mass indexed to body surface area and increased radial wall thickness which correlated with reduced longitudinal strain. This was the first study to perform late gadolinium enhancement (LGE) imaging and they found no evidence of focal intramyocardial fibrosis on late LGE imaging.^3^

In contrast, Yilmaz *et al*. performed the first prospective study of 37 patients with mitochondrial myopathy using a CMR protocol comprising cine, T2-weighted ‘‘oedema’’ imaging and T1-weighted LGE imaging.^18^ They found non-ischaemic late gadolinium enhancement in 32% (*n* = 12) of patients. Interestingly, 10/24 (42%) of the patients had a CPEO/KSS clinical syndrome and 2/3 (67%) patients with MELAS were LGE positive. The MERRF group did not have evidence of LGE, however, only 3 patients with this clinical syndrome were included in the study. All patients with CPEO/KSS had a LGE pattern of diffuse intramural enhancement in the left ventricular inferolateral segment. The T2-weighted assessment of the myocardium revealed high myocardial signal intensity suggestive of acute myocardial oedema in one (3%) patient with MELAS only.^18^ Following the study by Yilmaz, A small prospective study by Carteruccia *et al*. investigated 15 patients with MERRF harbouring the m.8344A > G mutation.^19^ In contrast to the Yilmaz study, this larger cohort of MERRF patients did demonstrate a similar pattern of LGE to that found in the CPEO/KSS and MELAS groups with 3/15 patients (20%) showing a pattern of subepicardial and intramural LGE predominantly involving the basal inferolateral segment of the left ventricle.^19^ In the same year a small prospective study used cardiac MRI comprising cine and LGE imaging in 33 patients with the CPEO or KSS clinical syndromes.^20^ They found that 8/33 patients (24%) demonstrated impaired LVEF and 10/33 patients (30%) demonstrated non-ischaemic LGE with a pre- dominantly intramural pattern, mostly confined to the basal LV inferolateral wall. Interestingly only 3 (9%) patients showed presence of LV hypertrophy. This is consistent with previous studies that demonstrated that KSS in particular can present with either LVH or a dilated cardiomyopathy phenotype.^21,22^ Barth syndrome is a rare mitochondrial myopathy syndrome that can also present with a dilated cardiomyopathy phenotype, usually in early childhood.^23^ Case reports have demonstrated cardiac MRI findings including dilated LV cavity size, reduced ejection fraction and hypertrabeculation, similar to echocardiography studies as well as LGE imaging showing diffuse fibrosis of the lateral LV wall.^24^

The largest study to date was performed by Florian *et al*., who prospectively investigated 64 patients with mitochondrial myopathy and 25 controls to characterize the prevalence and pattern of cardiac abnormalities and to test the additional diagnostic value of CMR in this patient population.^25^ They found that 53% of the cohort had at least one abnormal MRI finding defined as unexplained LVH (33%), reduced LV ejection fraction (28%) and/or LGE present in at least one myocardial segment (33%). Compared to controls, the total cohort of mitochondrial myopathy patients had significantly lower LV end-diastolic volumes (*P* = 0.028) and increased maximal wall thickness (*P* = 0.005) with significantly more frequent LV hypertrophy (*P* = 0.016) and higher concentricity as expressed by the ratio of LV mass to end-diastolic volume (*P* < 0.0001). Regarding LGE presence, 30% (*n* = 19) of the mitochondrial myopathy patients showed non-ischaemic intramural or subepicardial LGE in at least one myocardial segment, while no patients in the control group had evidence of non-ischaemic LGE (*P* = 0.001). This significant difference was primarily due to the high LGE prevalence in the MELAS (*n* = 8, 73%) and CPEO/KSS (*n* = 10, 30%) groups. They also described a slight difference in the LGE patterns between the CPEO/KSS and MELAS/-like groups. The CPEO/KSS group tended to have either intra-mural or subepicardial LGE involving the basal to mid inferolateral segments, whereas the MELAS/-like group displayed a more heterogeneous distribution and extent of LGE.

A mitochondrial DNA variant (m.4300A > G) has recently been found that causes isolated cardiac disease without systemic involvement, meaning red flags prompting mtDNA analysis may be absent. CMRI can be particularly important to help decide if additional genetic testing is required. Case series have described cardiac MRI findings including severe diffuse left ventricular wall hypertrophy without outflow obstruction, reduced left ventricular function, extensive fibrosis and progressive remodelling to left ventricular dilation and systolic dysfunction.^26–28^ Data on the median age at diagnosis among patients with the m.4300A > G mitochondrial DNA variant remains limited. This variant was first reported in 1995 in a male patient who presented with inherited hypertrophic cardiomyopathy at 20 years of age.^29^ A recent study investigated five families harbouring the m.4300A > G mtDNA variant and found the affected individuals were aged 16, 22, 24, 25, 40, 40, 47, and 67 years, with a median age of 32 years at diagnosis.^27^ Despite the relatively consistent cardiac phenotype, variability in age of onset and disease severity suggests that nuclear genetic modifiers, sex-specific factors, and secondary mitochondrial stressors may influence clinical expression in patients with this variant.^27^

The most recent study published in 2025 by Stevesandt *et al*. examined cardiac manifestations with CMRI in 11 adult patients with MELAS syndrome harbouring m.3243A < G-mutation.^30^ They also found 6/11 patients (55%) had evidence of non-ischaemic late gadolinium enhancement on cardiac MRI. Interestingly two patients had reduced ejection fraction in the range of 40–50% and one 45 year old male patient had evidence of severe cardiac involvement with an ejection fraction below 30% and extensive subepicardial and mid wall LGE in the inferior, anterior and septal segments of the left ventricle.^30^

Although there are differences in the CMR findings in mitochondrial cardiomyopathy based on the specific syndrome and affected gene, the most common CMRI findings include increased maximal LV wall thickness and LV mass, reduced end-diastolic volumes, and non-ischaemic subepicardial and midwall LGE, that can affect any segment but most commonly affecting the basal inferolateral or lateral wall (*Table 2*).^3,16–20,25,30^

### Clinical utility of cardiac MRI in mitochondrial cardiomyopathy

Early diagnosis of cardiomyopathy in mitochondrial disease may be difficult using conventional non-invasive methods such as echocardiography as this technique primarily shows non-specific LVH, with wall motion abnormalities only occurring at later stages of the disease course.^18^ For example Yilmaz *et al*. performed both echocardiography and cardiac MRI in 37 patients with mitochondrial myopathy and found that in 8/37 (22%) patients, LGE imaging revealed myocardial damage in the absence of any functional abnormalities. Performing just conventional echocardiography, which does not allow detection of intramyocardial tissue abnormalities, would have theoretically resulted in missing pathological findings in these patients. Similarly Florian *et al*. found CMR was superior to ECG and cardiac biomarkers in the detection of cardiomyopathy in their cohort of 64 patients with mitochondrial myopathy.^25^ CMR is superior to conventional echocardiography and allows detection of myocardial tissue damage even in the absence of functional abnormalities in patients with mitochondrial disorders due to its highly accurate measurement of LV volumes, mass, ejection fraction and its unique ability to assess tissue characterization. The evaluation of cardiac involvement in patients diagnosed with mitochondrial myopathy is clinically important, as evidence suggests a markedly increased mortality rate in individuals with mitochondrial myopathy and confirmed cardiomyopathy compared to those with mitochondrial disease without cardiac involvement.^1,6,14^ In a retrospective analysis of 101 paediatric patients with mitochondrial myopathy, Holmgren *et al*. reported a substantially higher incidence of cardiac-related mortality in those with cardiomyopathy (71%) compared to those without evidence of cardiomyopathy (26%). Furthermore, cardiac death was identified as the leading cause of mortality within this cohort.^14^ A large retrospective cohort study of 600 adults with genetically confirmed mitochondrial disease revealed that life-threatening major adverse cardiac events (MACE) occur in approximately 10% of patients over a 10-year follow-up period.^1^ In this study, MACE was defined as death or hospitalization due to heart failure, cardiac transplantation, sudden cardiac death, sustained ventricular tachycardia, or advanced atrioventricular block (third-degree or type 2 s-degree heart block). They found left ventricular hypertrophy and reduced left ventricular ejection fraction were independent predictors of MACE, underscoring the critical need for routine cardiac screening in patients with mitochondrial myopathy.^1^ Early detection of cardiac involvement before the onset of functional impairment, particularly through cardiac magnetic resonance (CMR) imaging, may facilitate the timely initiation of appropriate therapeutic strategies, risk stratification, family screening and timing of follow up. Potential treatment strategies could include early initiation of heart failure medications or device therapy, potentially improving patient outcomes, however this has not been proven in clinical trials.

**Figure 3:**
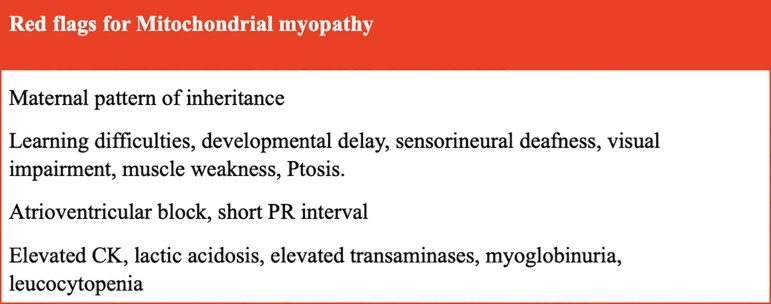
Red flags to raise the suspicious of mitochondrial myopathy.

**Figure 4:**
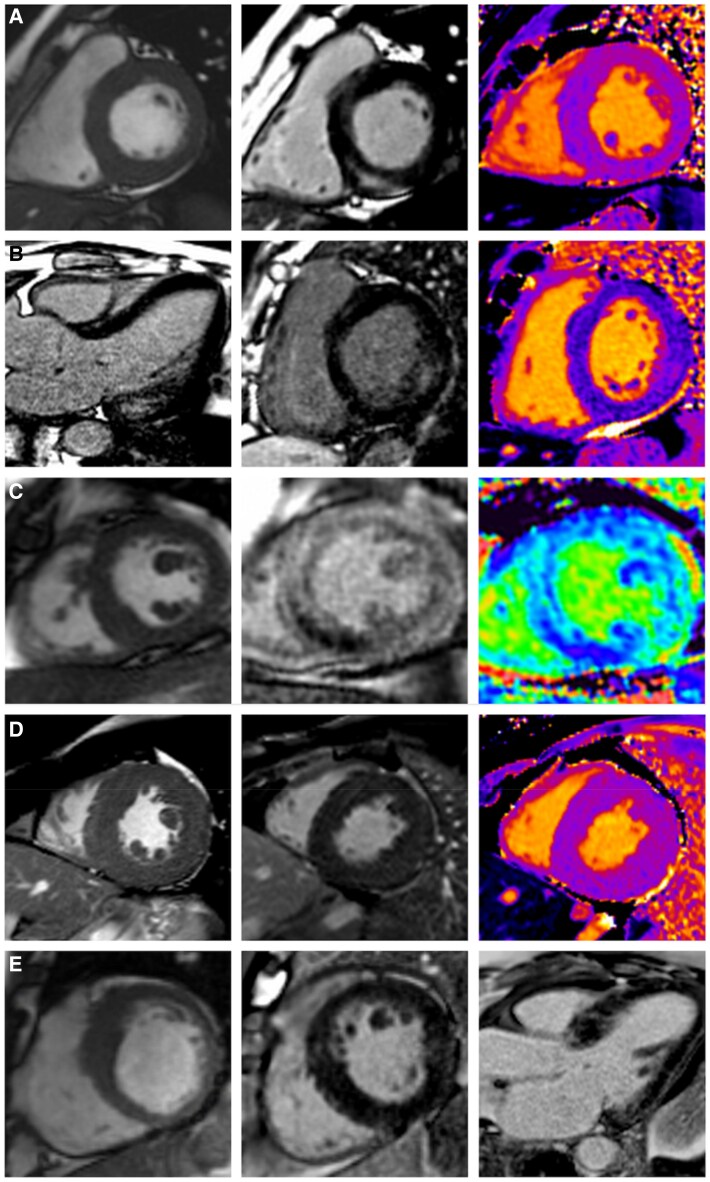
CMRI tissue characterization findings in different conditions causing LVH. Panel A: SSFP CINE, LGE and T1 map showing LVH (MWT 15mm) and basal inferior mid wall fibrosis in a patient with mitochondrial myopathy (m.3243A > G positive). Panel B: LGE 3 chamber and short axis images and T1 map values showing LVH (MWT 14mm) with basal inferolateral fibrosis and low T1 map values (850msec, 1.5T, MOLLI, reference 960–1050msec) in a 45yo male with Fabry's disease. Panel C: SSFP CINE, LGE and ECV map showing LVH (MWT 15mm) with diffuse subendocardial and transmural LGE and elevated ECV (41%) in a patient with AL-amyloidosis. Panel D: SSFP CINE, LGE and T1 map values showing LVH (MWT 16mm) with no evidence of focal or diffuse fibrosis in a 25yo with Friedreich's ataxia. Panel E: SSFP imaging, short axis and 3 chamber LGE imaging showing asymmetrical LVH (MWT 15mm) in a 39yo female with *MYBPC3* gene positive HCM.

CMR also has a valuable role to play in the assessment of patients with LVH of unclear cause, especially if pathogenic sarcomeric hypertrophic cardiomyopathy genes have not been identified and/or there are clinical red flags suggestive of a possible metabolic syndrome. Mitochondrial myopathy can be a challenging diagnosis to make, especially if one of the well documented clinical syndromes is not apparent. This is due to their rarity; clinical, biochemical, and genetic heterogeneity; their phenotypic overlap with other disorders; and their lack of specific biomarkers.^31^ As a consequence, patients are frequently misdiagnosed and consult, on average, more than eight physicians before the correct diagnosis is made.^31^ Cardiologists should be aware of the clinical red flags (*Figure 3*) and CMRI findings of mitochondrial cardiomyopathy as described in the section above, and how to differentiate from other causes of LVH on CMRI (*Figure 4*). For example sarcomere gene positive HCM often presents with asymmetric septal LVH and myocardial replacement fibrosis on LGE imaging in segments of maximal hypertrophy^33^; Amyloidosis presents with concentric LVH, significantly reduced global longitudinal strain with apical sparing, very high ECV and diffuse subendocardial LGE often with a basal predominance^34^; Fabry's disease displays concentric LVH with basal lateral fibrosis and reduced native T1 map values^35^; Aortic stenosis can present with LV concentric or asymmetrical remodelling, concentric or asymmetrical hypertrophy, or eccentric hypertrophy, as well as both focal and diffuse fibrosis demonstrated on LGE, T1 mapping and ECV imaging in response to chronic pressure overload^36^; Athletes can develop increased wall thickness often between 13–16mm, but usually do not have a reduction in LV cavity size, and maintain normal diastolic function, and ECV^37^; Friedreich's ataxia can have a very similar CMRI appearance to mitochondrial myopathy with LVH and midwall or subendocardial LGE with a basal predominance.^38^ Friedreich's ataxia is an autosome recessive trinucleotide repeat (GAA) expansion disorder caused by mutation in the FXN gene leading to deficiency of the mitochondrial protein frataxin.^38^ This condition does lead to mitochondrial dysfunction however is classified as a hereditary cerebellar ataxia rather than a mitochondrial myopathy. Its main clinical features include progressive ataxia, dysarthria, absent lower limb reflexes, upgoing plantar responses, and peripheral sensory neuropathy.^38^

**Table 2:** Pivotal CMRI trials in mitochondrial myopathy

First author, year	Study design, study population	No. of patients	Controls	Main result
Hollingsworth, 2012^16^	Case-control, m.3243A > G mtDNA mutation positive patients.	10	16 healthy age matched controls	Structural and functional cardiac abnormalities in all patients compared to controls. Reduced end systolic and end diastolic volumes, concentric LVH and reduced longitudinal shortening.
Lindroos, 2016^17^	Case-control, m.3243A > G mtDNA mutation positive patients.	14	13 controls matched for age, physical activity and body mass index.	Reduced end diastolic volume, reduced ejection fraction indexed to BSA, increased LV mass compared to controls.
Bates, 2013^3^	Case-control, m.3243A > G mtDNA mutation positive patients	22	22 age- and gender-matched control	Compared with control subjects, patients had an increased left ventricular mass index and LV mass to end-diastolic volume ratio. Longitudinal shortening was decreased. No patients displayed focal LGE.
Yilmaz, 2012^18^	Prospective cohort study. CPEO/KSS (*n* = 24), MELAS (*n* = 3), MERRF (*n* = 2), other (*n* = 8).	37	Nil	LGE found in 32% (*n* = 12) of patients. LGE more common in patients with CPEO/KSS and MELAS. The MERRF group did not have evidence of LGE. Elevated T2 stir imaging in one patient with MELAS. In 22% of patients.
Catteruccia, 2015^19^	Prospective cohort study, A8344G ‘‘MERRF’’ mtDNA mutation positive patients	15	Nil	3/15 patients (20%) LGE positive. Pattern of subepicardic and intramural LGE predominantly involving the basal inferolateral segment of the left ventricle.
Florian, 2015^25^	Prospective cohort study, CPEO/KSS (*n* = 33), MELAS/-like (*n* = 11), MERRF (*n* = 3), other (*n* = 17).	64	25 healthy individuals matched for age, gender and cardiovascular risk factors	Compared to controls, patients had increased LV mass index, increased LV wall thickness, reduced end systolic and end diastolic volumes. LVH was most common in the MELAS/-like group.
				30% of the CPEO/KSS and 73% of the MELAS/-like group were LGE positive.
Stoevesandt, 2025^30^	Cross sectional study, m.3243A < G mtDNA mutation and confirmed diagnosis of MELAS syndrome.	11	Nil	6/11 patients (55%) had non-ischaemic LGE. 3/11 patients (27%) had reduced LVEF.

**Figure 5:**
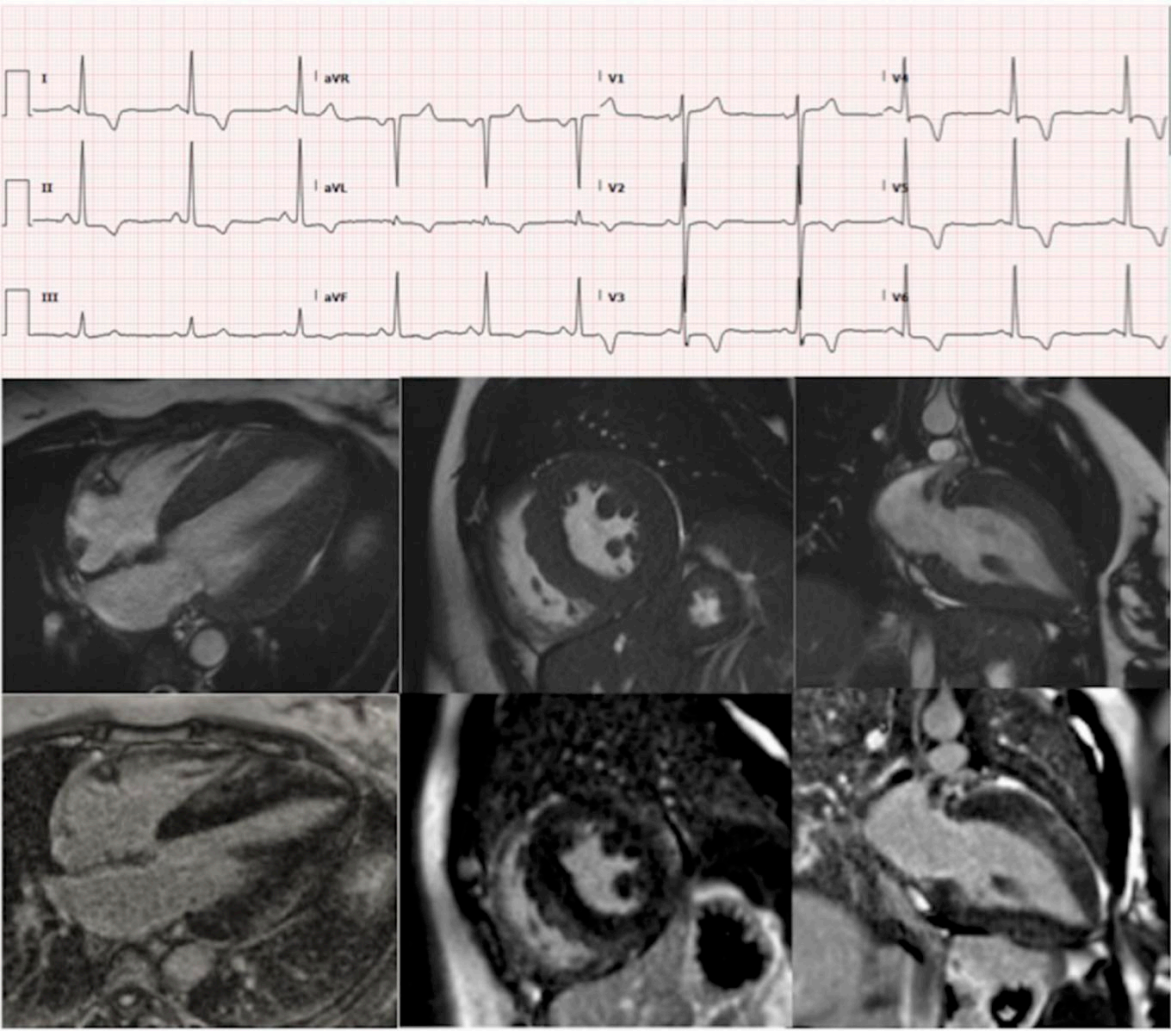
ECG and cardiac magnetic resonance (CMR) images in a 35year old female found to have mitochondrial myopathy (m.3243A > G mutation). Past medical history included well-controlled hypertension diagnosed at 19 years, bilateral deafness attributed to parotiditis, repeat miscarriages (five), and gestational diabetes. CMR revealed symmetrical/concentric left ventricular hypertrophy maximum LV wall thickness 16 mm and extensive fibrosis with subepicardial distribution. (upper row end-diastolic cine images, lower row late gadolinium enhancement images). Adapted with permission from Lopes, Luis R., *et al*. “Iterative reanalysis of hypertrophic cardiomyopathy exome data reveals causative pathogenic mitochondrial DNA variants.” *Circulation: Genomic and Precision Medicine* 14.3 (2021): e003388.^32^

Multiple case reports and case series in the literature have demonstrated the diagnostic value of CMRI in patients presenting with significant LVH and an unclear diagnosis.^39–42^ For example Partington *et al*. describe the case of a 29 year-old female who presented with an unknown cause of heart failure and concentric LVH with a maximal wall thickness of 17mm on echocardiogram. CMRI findings included subepicardial and midwall LGE and signal hyperintesity on T2W imaging raising the possibility of mitochondrial cardiomyopathy and prompted a thorough family history. This revealed a maternal history of diabetes, seizures, and stroke-like syndrome leading to the provisional diagnosis of mitochondrial cardiomyopathy which was confirmed with muscle biopsy and genetic testing.^40^ Similarly a case series by Lopes *et al*. describe 3 patients in whom a primary mitochondrial disease diagnosis had not previously been suspected. CMRI demonstrated severe LVH and extensive fibrosis with subepicardial distribution which was unusual for sarcomeric HCM (*Figure 5*). Reanalysis of whole-exome sequencing data for mtDNA mutations was performed which revealed the m.3243A > G variant in 2 patients with 37% and 11% blood load, and the m.3460G > A variant with homoplasmic blood levels.^32^ A recent study by Chung *et al*. performed comprehensive genetic and CMRI analysis on 133 patients with a diagnosis of hypertrophic cardiomyopathy. Interestingly they found 24 patients had mitochondria related nDNA or MtDNA pathogenic variants with 12/24 of these patients (50%) having evidence of LGE on CMRI.^43^ Compared to patients with a sarcomere gene variant, the mitochondrial gene variant group had significantly less LGE in the septal segments, which is in keeping with the majority of the literature describing subepicardial and midwall LGE mainly involving the lateral segments in patients with mitochondrial cardiomyopathy.

These studies along with the case we described earlier illustrate the clinical utility of cardiac MRI above and beyond echocardiography to help in the diagnosis of mitochondrial cardiomyopathy through its unique ability of tissue characterization.

### Current recommendations

An optimal CMRI protocol for assessing cardiac involvement in mitochondrial disease patients includes T2 black blood axial stack to assess anatomy. Two, three, four chamber and short axis SSFP CINE imaging for left and right ventricular volumes, mass, ejection fraction and global longitudinal strain analysis. Pre and post contrast T1 mapping and late gadolinium enhancement imaging for the assessment of myocardial necrosis, scar and fibrosis.

The recent ESC cardiomyopathy guidelines encourage multimodality imaging for morphological and functional characterization of cardiomyopathy to determine the phenotype and recommend CMR in the initial evaluation of patients with cardiomyopathy with a level 1 class B recommendation.^12^ However, the widespread use of CMRI in the assessment of undiagnosed LVH and in screening for cardiac involvement in mitochondrial myopathy is limited by access and funding in many countries. The 2017 consensus-based recommendations for optimal management and care for patients with primary mitochondrial disease published by the Mitochondrial Medicine Society recommend cardiac screening with echocardiography and ECG at initial diagnosis and at 1–2 year intervals, with cardiac MRI only recommended on an as needed basis depending on symptoms or disease type.^12^ We advocate that a baseline CMR should be considered in all patients with mitochondrial myopathy and an abnormal ECG, echocardiogram, or Holter until more data is available.

### Future directions

Large prospective studies using cardiac MRI with extended follow-up are warranted in patients with mitochondrial myopathies, especially those with rare clinical syndromes, to further elucidate the incidence and cardiac phenotype in this population. Future investigations should also consider integrating cardiac MRI, particularly late gadolinium enhancement imaging, into risk prediction models to enhance stratification of major adverse cardiac events such as heart failure and arrhythmia in patients with mitochondrial cardiomyopathy. This could be achieved by integrating clinical variables, cardiac biomarkers, and electrocardiographic data with cardiac magnetic resonance imaging, particularly utilizing late gadolinium enhancement, which has been shown to predict sudden cardiac death in patients with hypertrophic cardiomyopathy.^44^ The incorporation of clinical, ECG, and imaging data, potentially enhanced by artificial intelligence, may ultimately lead to superior risk stratification models for these patients.

Future studies could also consider incorporating oxygen-sensitive cardiovascular magnetic resonance techniques to further elucidate myocardial pathophysiology in mitochondrial myopathies, in which impaired oxidative phosphorylation is a central feature. Oxygenation-sensitive cardiac magnetic resonance imaging (OS-CMR) is a non-invasive, contrast-free method for mapping and monitoring myocardial oxygenation, exploiting paramagnetic deoxyhaemoglobin as an intrinsic contrast mechanism via the blood oxygen level–dependent (BOLD) effect. OS-CMR has emerged as a promising tool for detecting changes in myocardial oxygenation and has been applied across a range of cardiac diseases, providing novel insights into the underlying pathophysiology of various cardiomyopathies.^45^ These advanced techniques may enable the detection of subtle or regional alterations in myocardial oxygenation and function before overt structural changes occur, potentially improving early diagnosis and risk stratification. Incorporating OS-CMR into longitudinal studies may further clarify the contribution of mitochondrial dysfunction to disease progression and response to emerging therapies.

In addition, future CMRI studies could evaluate whether early treatment, with either novel targeted therapies or agents such as angiotensin-converting enzyme, can prevent or slow the progression of mitochondrial cardiomyopathy, as early introduction of perindopril has been shown to delay the onset of systolic dysfunction in other forms of genetic cardiomyopathy.^46^

## Conclusion

Cardiac involvement is a critical yet often under-recognized component of mitochondrial myopathy, with significant implications for patient prognosis. Cardiovascular magnetic resonance imaging not only aids in the assessment of cardiac involvement in known cases but also plays a valuable diagnostic role in patients presenting with unexplained left ventricular hypertrophy (LVH). As our understanding of mitochondrial disease evolves, integrating advanced imaging with molecular diagnostics will be essential for early detection, accurate diagnosis, and improved clinical outcomes.

## Data Availability

No new data were generated or analysed in support of this research.

## Lead author biography

Dr. Rhys Gray is an Australian-trained cardiologist currently serving as a Senior Clinical Fellow in Advanced Cardiac Imaging and Inherited Cardiac Conditions at Barts Heart Centre in London. His clinical and research interests include multi-modality cardiac imaging, inherited cardiomyopathies and cardio-oncology. Dr Gray holds EACVI level 3 accreditation in cardiac MRI, as well as accreditation in echocardiography and Cardiac CT. He is passionate about integrating multi-modality cardiac imaging with clinical cardiology to enhance patient care.
